# WHEN FAMILIES DISAGREE: CORRELATES AND STRESS PROCESS OUTCOMES OF FAMILY CAREGIVER DISAGREEMENT

**DOI:** 10.1093/geroni/igac059.1645

**Published:** 2022-12-20

**Authors:** Amanda Leggett, Hyun Jung Koo, Hannah Lee, Elaina Baker

**Affiliations:** Wayne State University, Ypsilanti, Michigan, United States; University of Michigan, Ann Arbor, Michigan, United States; University of Michigan, Ann Arbor, Michigan, United States; University of Michigan, Ann Arbor, Michigan, United States

## Abstract

Dementia caregiving is a family affair, yet prior research suggests that families often disagree about care provision. We explore correlates of such disagreements and how disagreements relate to stress process outcomes of care. The current study examines 552 family caregivers for Medicare-eligible older adults with dementia from the 2017 National Study of Caregiving. We consider demographic and care characteristics as predictors of family disagreement in a multinomial logistic model, and family disagreement as the key predictor of caregiving overload, emotional difficulty, and gains in linear regressions. Females had greater odds of disagreement, while those with friends and family to talk to and providing assistance with household care activities had lower odds of disagreement. Family disagreement was significantly associated with overload (B=0.95, p<.001) and emotional difficulty (B=0.45, p<.05), but not gains. Interventions to help families manage care disagreements and coordinate care may have great impact on caregiver well-being.

